# Randomized Trial of Artesunate+Amodiaquine, Sulfadoxine-Pyrimethamine+Amodiaquine, Chlorproguanal-Dapsone and SP for Malaria in Pregnancy in Tanzania

**DOI:** 10.1371/journal.pone.0005138

**Published:** 2009-04-08

**Authors:** Theonest K. Mutabingwa, Kandi Muze, Rosalynn Ord, Marnie Briceño, Brian M. Greenwood, Chris Drakeley, Christopher J. M. Whitty

**Affiliations:** 1 Department of Infectious and Tropical Diseases, London School of Hygiene & Tropical Medicine, London, United Kingdom; 2 National Institute for Medical Research, Dar-es-Salaam, Tanzania; 3 Muheza Designated District Hospital, Muheza, Tanga Region, Tanzania; 4 Muhimbile Medical Centre, Dar-es-Salaam, Tanzania; 5 Department of Genome Sciences, University of Washington, Seattle, Washington, United States of America; Royal Melbourne Hospital, Australia

## Abstract

**Background:**

Malaria in pregnancy is serious, and drug resistance in Africa is spreading. Drugs have greater risks in pregnancy and determining the safety and efficacy of drugs in pregnancy is therefore a priority. This study set out to determine the efficacy and safety of several antimalarial drugs and combinations in pregnant women with uncomplicated malaria.

**Methods:**

Pregnant women with non-severe, slide proven, falciparum malaria were randomised to one of 4 regimes: sulfadoxine-pyrimethamine [SP]; chlorproguanil-dapsone [CD]; SP+amodiaquine [SP+AQ] or amodiaquine+artesunate [AQ+AS]. Randomisation was on a 1∶2∶2∶2 ratio. Women were admitted for treatment, and followed at days 7, 14, 21, 28 after the start of treatment, at delivery and 6 weeks after delivery to determine adverse events, clinical and parasitological outcomes. Primary outcome was parasitological failure by day 28.

**Results:**

1433 pregnant women were screened, of whom 272 met entry criteria and were randomised; 28 to SP, 81 to CD, 80 to SP+AQ and 83 to AQ+AS. Follow-up to day 28 post treatment was 251/272 (92%), and to 6 weeks following delivery 91%. By day 28 parasitological failure rates were 4/26 (15%, 95%CI 4–35) in the SP, 18/77 (23%, 95%CI 14–34) in the CD, 1/73 (1% 95%CI 7–0.001) in the SP+AQ and 7/75 (9% 95%CI 4–18) in the AQ+AS arms respectively. After correction by molecular markers for reinfection the parasitological failure rates at day 28 were 18% for CD, 1% for SP+AQ and 4.5% for AQ+AS. There were two maternal deaths during the trial. There was no apparent excess of stillbirths or adverse birth outcomes in any arm. Parasitological responses were strikingly better in pregnant women than in children treated with the same drugs at this site.

**Conclusions:**

Failure rates with monotherapy were unacceptably high. The two combinations tested were efficacious and appeared safe. It should not be assumed that efficacy in pregnancy is the same as in children.

**Trial Registration:**

ClinicalTrials.gov NCT00146731

## Introduction

Malaria in pregnancy is an important preventable cause of maternal and perinatal morbidity and mortality [1], [2], and contributes substantially to maternal morbidity in Tanzania and elsewhere [3]. Pregnant women are at increased risk of clinical disease compared with non-pregnant women [4], [5]. In pregnancy, women semi-immune to malaria carry substantial risks of severe maternal anaemia and low-birthweight which is greatest in the first pregnancy, and the malaria may be asymptomatic [6]–[8]. In contrast, non-immune pregnant women, and possibly women with HIV infection, are at a greater risk of premature delivery, hypoglycaemia, severe anaemia, pulmonary oedema and maternal death^2^.

The risks of malaria in pregnancy are therefore substantial both to the mother and foetus. Using an effective antimalarial drug for prevention and treatment is essential. Understandable concerns are raised however by using any new drug in pregnancy; older drugs have a better known safety profile in pregnancy, but are likely over time to become less effective due to the emergence and spread of drug resistance. Although there are no human data to suggest that artemisinins are teratogenic, animal data for use of artemisinins early in pregnancy have raised concerns of teratogenicity [9]. Traditional exclusion of pregnant women from clinical trials has led to limited data on safety and efficacy of artemisinin based combinations considered for general deployment especially in Africa. Current practice of deriving malaria treatment policies for pregnancy from data reporting efficacy of drugs in children is inappropriate. Therefore, generating data on the efficacy and safety of antimalarials in pregnancy is a priority [10], [11]. This trial was therefore designed to assess the efficacy and tolerability of two antimalarial drug combinations and one novel form of monotherapy in pregnant Tanzanian women.

Sulfadoxine-pyrimathamine (SP) has been assessed for safety in pregnancy, is now recommended for intermittent preventive treatment in pregnancy (IPTp), and remained Tanzanian national policy for treatment until November 2006 and for Intermittent Preventive Therapy in pregnancy in most countries (IPTp) to date [12], [13]. Amodiaquine (AQ) has been given to many hundreds of women in pregnancy in Africa and elsewhere, either inadvertently or deliberately, and studies have demonstrated no teratogenicity, although formal safety data are sparse [14], [15]. The WHO does not consider that the combination of amodiaquine+artesunate in pregnancy is contraindicated but evidence of its safety, in common with other combinations, is sparse [16]. Chlorproguanil-dapsone (CD) has proved effective as monotherapy in children in the Muheza district (present study area) even when sulfadoxine-pyramethamine was failing [17]. This drug provided a possible choice of treatment for malaria in pregnancy at the time this trial was planned. Experience with co-administered CD in pregnancy is limited to its use as a single dose (chlorproguanil 1.2 mg/kg and dapsone 2.4 mg/kg as a single dose), where it appeared safe [18]. Proguanil has been recommended for use as a safe antimalarial in pregnancy for many years. Experience with dapsone treatment during pregnancy used in Hansen's disease (leprosy) and in other pregnancy related conditions is reassuring [19]. Subsequent to this trial CD has been withdrawn due to safety concerns in the CD-artesunate (CDA) combination [20].

Despite concerns from animal studies both artesunate and artemether have been given to many pregnant women (often inadvertently), and current published data demonstrate no evidence of human teratogenicity [21]. The WHO recommends artemisinin derivatives can be used for malaria treatment during the second and third trimesters of pregnancy in all settings and in the first trimester where multi-drug resistance prevails and the benefits outweigh the risks [22]. Whilst most of the data from treatment doses is from Asia, 287 pregnant women in the Gambia given a single dose of SP-artesunate had no increased rate of adverse birth outcomes [23].

The present study compared the efficacy, tolerability and safety of standard SP-monotherapy to CD that had been registered in UK at the design of the study and to two drug combinations for which good efficacy data was available from East Africa. AQ+SP has proved effective both in Ugandan and Tanzanian children [24], [25], and in pregnancy in West Africa [26] and in some settings proves better than artemisinin-based combinations [27]. AQ+AS is first-line treatment in Zanzibar, and there is data on its efficacy from several East African sites suggesting that it is significantly more efficacious than amodiaquine monotherapy [21], [28], [29].

## Methods

This open-label study was conducted among pregnant women who attended Muheza Designated District Hospital (Muheza DDH). The lowlands of Muheza district experience hyperendemic to holoendemic malaria. The day 28 parasitological failure rates to AQ monotherapy in a recent effectiveness trial in children under 5 years was 76%, with the comparative rates of 61% and 40% for AQ+SP and AQ+AS^21^. Throughout the study period SP (defined as monotherapy for the purposes of this paper) was national first-line treatment for malaria, and this was taken as the comparator arm for this study.

Pregnant women with mild-moderate, slide proven, falciparum malaria were recruited from the Antenatal wing (ANC) of the Reproductive and Child Health (RCH) clinic at Muheza Designated District Hospital. Pregnant women from Muheza Township and surrounding villages attend this clinic for their medical care. Nurses at the RCH identified all febrile pregnant women with a fever or recent history of fever (within 48 hours), symptoms compatible with anaemia or malaria and referred them to the study team. All referrals were re-interviewed and examined by a medical officer from the study team to exclude concomitant infection(s). Duplicate thick and thin blood smears were Giemsa stained and examined microscopically for malaria parasites.

Inclusion criteria were pregnancy with *either* a positive blood smear for *P.falciparum* with at least 800 asexual parasites/µL in an asymptomatic woman *or* any of the following symptoms within 2 days prior to consultation; history of fever; headache, vomiting, chills/rigors, and/or any of the following signs: temperature ≥37.5°C and <39.5°C, Hb≥7 and <9 g/dl) together with *P.falciparum* parasitaemia at any density. Additionally, all cases had to be 14–34 gestation weeks pregnant on the day of attending the clinic, have a viable foetus defined by the presence of foetal heartbeat by sonicaid or pinnard, able to take drugs orally, able to attend follow up clinic, and gave written informed consent to participate or a finger-print witnessed consent for women unable to read.

The main exclusion criteria were; severe and complicated forms of malaria [30], pregnancy in the first trimester or >34 gestation weeks (because they had a high chance of delivering during the 28 day follow-up period), mixed plasmodial infection, complicated pregnancy e.g. signs/symptoms of toxaemia, 2 or more abortions or stillbirths, presence of concomitant disease masking assessment of the response to treatment, intake of drugs contraindicated in pregnancy or drugs with effective antimalarial activity within the last 2 weeks, multiple gestation pregnancies, mother aged 38 years or above. Withdrawal criteria were withdrawal of consent, appearance of other species of *Plasmodium* or major protocol violation.

Women who met the inclusion criteria were randomised to one of 4 regimes: three tablets of sulfadoxine-pyrimethamine (500 mg sulfadoxine/25 mg pyrimethamine per tablet) orally at once [SP] in line with the national policy; chlorproguanil-dapsone (1.2 mg/kg and 2.4 mg/kg respectively for 3 days) [CD]; SP 3 tablets once+amodiaquine (10 mg/kg for 3 days) [SP+AQ]; amodiaquine (10 mg/kg for 3 days)+artesunate (4 mg/kg for 3 days) [AQ+AS]. Randomisation was on a 1∶2∶2∶2 ratio for SP, CD, SP+AQ and AQ+AS to maximise information about the drugs whose use in pregnancy is less known; it was assumed the difference in outcome would be greatest for SP compared to other arms so the size of this arm could be smaller. Randomisation was in blocks of random sizes, and conducted in London using Stata 7. Treatment allocations were placed in a sealed opaque envelope, with pregnant women picking their own envelope. Patients were allocated a study number sequentially, and after consenting to participate, participants picked an envelope in front of the attending clinician. Opening the envelope constituted entry to the trial and analysis was conducted on that basis (defined as analysis of all cases in which there was an outcome, irrespective of actual treatment given).

All women were admitted to a ward dedicated to research for the first three days to facilitate supervised drug administration and to monitor clinical response and adverse events. Drugs were administered by study nurse-midwives employed by the project. After each administration, the patient was observed for 45–60 minutes. The dose was repeated if vomiting occurred within the observation period. Vomiting the second dose was registered as an adverse event and led to withdrawal from the study. Such cases were treated with parenteral quinine. Patients were treated for symptoms with standard medications e.g. paracetamol for fever. Women continued to receive routine antenatal medicaments of iron supplements, folic acid (5 mg), and tetanus toxoid (TT) given by the RCH. In addition to daily clinical observations and laboratory tests, foetal viability (presence of foetal heartbeat) was monitored daily during admission using a Doppler machine, and at each follow-up visit.

Adverse events were classified by severity and potential causal relationship to study drugs. All serious adverse events (SAEs) were independently investigated by a local safety monitor. Subjects with an AE were followed up until the condition had disappeared or stabilized.

Parasite counts on Giemsa-stained blood films were performed daily during admission, repeated on days 7, 14, 21 and 28, and on any other day(s) of complaints. Counts were made against 200 white blood cells (WBC) on a thick blood smear. All slides had a second reading done in an independent research laboratory. Discordant results were read by a third reader, with the majority taken as the definitive outcome. All microscopists were blind to treatment allocation. A separate read for gametocytes was undertaken counting against 500 white blood cells. To quantify the effect of treatment on gametocyte carriage, we determined the area under the curve (AUC) of gametocyte density over time which incorporates both the magnitude and the duration of gametocyte carriage [31].

Blood samples for haematology and clinical chemistry, and in anaemic women stool microscopy for intestinal helminths were obtained on admission. Blood and urine samples were repeated at day 3 and where indicated at day 7. Haemoglobin, total and differential white blood cell count, platelet count, creatinine, total bilirubin, alanine aminotransferase (ALT) and albumin were measured (using CBC machine for haemogram and Reflectron for biochemistry) on days 0, 3 and 7, and whenever else indicated. Pre-test counselling for HIV-testing was undertaken, and where consent was given an HIV test was performed. HIV-positive mothers were referred to the HIV care unit of Muheza DDH for counselling and for consideration of antiretroviral drugs.

On discharge from the ward, patients were followed up on days 7, 14, 21 and 28 post initiation of treatment and at any time they felt unwell before day 28. At the end of each clinic, members of the study team followed all non-attendees to their homes to establish and record reasons for non-attendance and to collect a blood smear. Patients with either early or late treatment failure following treatment with any of the 4 study regimens were treated with quinine 10 mg/kg 8 hourly for 7 days as rescue therapy.

Birth outcome and Dubowitz assessment were recorded for all deliveries taking place at the hospital. Mothers and their newborns attended follow up clinics 6 weeks after delivery. All non-attendees (whether delivered at Muheza DDH or not) were followed up at home. Assessment at this time included a further check of the newborn by the paediatrician for any abnormality that may have been missed at birth or for any serious problem that may occur after birth such as kernicterus. Whenever the mother had moved from the study area, all possible efforts were made to ascertain birth outcome verbally from close relatives. A DSMB reviewed all SAEs, which were notified as they occurred.

Blood for PCR was collected on glass-fibre membranes from all patients at enrolment and at each follow-up. The polymorphic repetitive regions were amplified by nested-PCR for block 3 of msp2 [32]. Using the template of the first PCR reaction, allele-specific primer pairs was used to test for the presence of the allelic variants from FC27 and IC of the families of the msp2 region. Amplification patterns of the various allelic families in DNA samples from day 0 were compared to other samples from the same patient when parasitaemic. If the allelic family(ies) amplified on day 0 included those which were identical in size to those amplified during a subsequent episode, then the patient was classified as carrying a recrudescent infection.

The primary end-point of the trial was parasitological failure by day 28. This was defined as any of: a need for rescue treatment due to clinical deterioration defined by altered sensorium, seizures, persistent vomiting, renal impairment, respiratory distress, a fall in Hb below 7 g/dl, or in cases where the initial haemoglobin dropped 20% or more from baseline Hb, at any time during admission; persistence of fever with parasitaemia on day 3; increased parasite density on day 2 or 3 compared with baseline density; failure to clear parasites on day 7; rescue medication for recurrent malaria before day 28; slide parasite positivity at day 14, 21 or 28.

Major secondary endpoints were: clinical failure by day 28 (parasitological in the presence of symptoms compatible with malaria), parasitological or clinical failure by day 14, incidence of foetal death during treatment, defined as absence of foetal heartbeat assessed by Doppler; change in haemoglobin from baseline on day 14; incidence of perinatal and neonatal mortality, assessed 4–6 weeks after due date of delivery; clinically apparent neonatal abnormality 4–6 weeks after due date of delivery; preterm delivery and other adverse events during treatment.

Initially the study was powered to detect a 4 fold difference in treatment failure between SP+amodiaquine and amodiaquine+artesuante groups (8% vs 2%) with 95% precision and 80% power, which would require a samples size of 80 women in the SP+placebo group and 240 women in each of the other three groups. Vigorous measures to protect pregnant women in the district from malaria on a general background of reduced transmission of malaria in this area fortunately led to substantial reductions in the number attending the antenatal clinic with clinical malaria. The data from this and other sites was reviewed on October 2004 and it was decided that given the absence of other data, the question was important enough and of sufficient public health priority that a trial able to detect a larger difference would still be of public health importance. A revised sample size was calculated to detect a difference from 1% (the best likely failure rate in any arm) and 15% (above which no drug could be deployed). This gave a sample size of 72 in each arm when α was .05 and β 0.8. The unbalanced sample size (1∶2∶2∶2) was by this time established and could not be revised retrospectively although the statistical rationale for it was not present with the revised design.

Data were double entered into Microsoft Access, and analysed using Stata 8. The analytical plan was finalised before the analysis was undertaken. For primary and major secondary outcomes, proportions with confidence intervals were calculated. Odds ratios were calculated for the difference between all arm and the best and worst arms for parasitological failure unadjusted, and adjusted for the predefined risk factors age, parity, HIV serostatus, initial parasitaemia and initial haemoglobin.

Ethical permission was granted by the ethics committees of the National Institute for Medical Research, Tanzania, and the London School of Hygiene & Tropical Medicine and conducted in accordance with the Declaration of Helsinki. All participants gave written informed consent (or witnessed where whey could not read). The trial was monitored by an independent external clinical monitor and was prospectively registered on ClinicalTrials.gov No. NCT00146731. The protocol for this trial and supporting CONSORT checklist are available as supporting information: see CONSORT S1 and Protocol S1.

## Results

**Figure 1:**
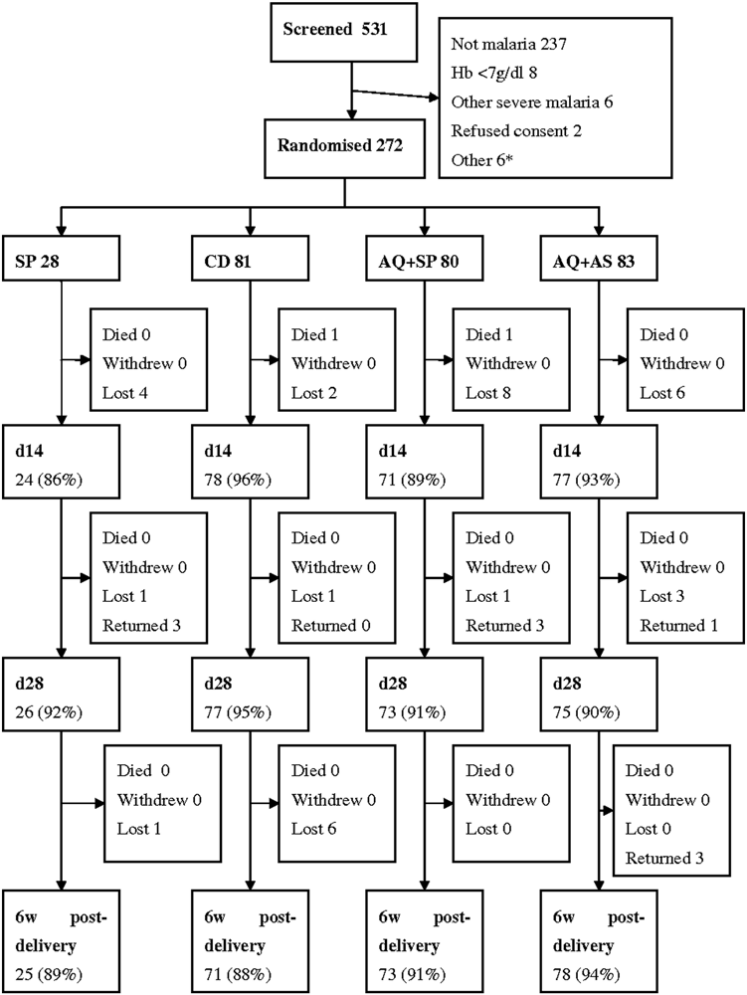
Flow through the trial. Data not adjusted for PCR correction. *Other includes living out of study area, multiple pregnancy, masking disease. Returned: returned to study area; no intercurrent treatment (d0–d28). SP = sulfadoxine-pyrimethamine AQ = amodiaquine CD = chlorproguanil-dapsone AS = artesunate. d14 = day 14 post-randomisation d28 = day 28 days post-randomisation; 6w post-del = 6 weeks post delivery.

The trial ran from Jan 2004–Sept 2006. 1433 pregnant women were screened, of whom 272 were enrolled, 28 to the SP, 81 to the CD, 80 to the SP+AQ and 83 to the AQ+AS arms respectively. The slight variation from the planned 1∶2∶2∶2 randomisation was due to random variation in the smallest arm because the trial did not reach the originally planned sample size on which the randomisation was based. Reasons for exclusion and flow through the trial are outlined in Fig. 1. The patients were similar at baseline (Table 1) and the prevalence of markers associated with antifolate resistance was high in all baseline samples (DHFR 51I+59R+108N = 96.2%; DHPS S436+437G+540E = 92.0%). Follow-up to day 28 post treatment was 251/272 (92%), and to 6 weeks following delivery 91%. Almost all those lost to follow-up were confirmed as having moved out of the study area.

**Table 1:** Baseline characteristics in the four arms.

	SP	CD	SP+AQ	AQ+AS
Number	28	81	80	83
Median age in years (IQR)	21 (19–26)	21 (19–27)	20 (19–25)	21 (19–26)
Median gestation in months (IQR)	6 (5–8)	7 (6–8)	7 (5–7)	6 (5–7)
Mean haemoglobin g/dL (SD)	9.3 (1.3)	9.6 (1.2)	9.0 (1.3)	9.3 (1.3)
Median parasite count (/200 WBC)	184 (55–535)	106 (23–650)	25 (51–578)	181 (62–628)
Median days unwell	3	3	3	3
% with primary education	93	90	85	91
Gametocytes at presentation (%)	23%	20%	17%	12%
DHFR triple mutation; N = 131* (51I+59R+108N)	83.3% (10/12)	94.7% (36/38)	100% (46/46)	97.1% (34/35)
DHPS double mutation; N = 137 (S436+437G+540E)	92.3% (12/13)	89.2% (33/37)	97.9% (46/47)	85.0% (34/40)
DHFR Triple+DHPS double; N = 119	81.8% (9/11)	82.4% (28/34)	97.7% (42/42)	93.5% (29/31)
HIV test positive (%)	0/27	1/80 (1.3)	1/82 (1.2)	0/79
Primiparous** (%)	7/12 (58)	26/42 (62)	14/38 (39)	20/34 (59)

* Some samples for PCR were lost in transit from field to laboratory.

** Parity was not recorded for the initial study participants.

**Figure 2:**
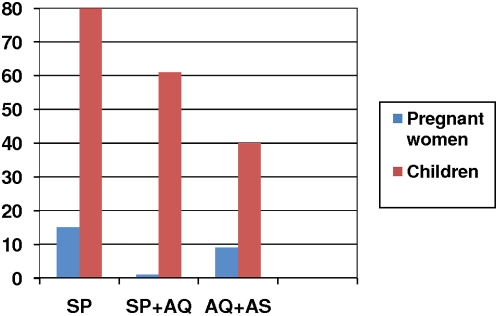
Day 28 parasitological failure rate (%), in pregnant women compared with children from the same site.* Data on children from Mutabingwa TK et al. Amodiaquine alone, amodiaquine+sulfadoxine-pyrimethamine, amodiaquine+artesunate, and artemether-lumefantrine for outpatient treatment of malaria in Tanzanian children: a four-arm randomised effectiveness trial. Lancet. 2005;365:1474–80. Unadjusted for PCR correction, study in children >5 years 2003–4.

By day 14, the parasitological failure rates (including both symptomatic and asymptomatic cases) were 1/24 (4%) in the SP, 1/78 (1.3%) in the CD, 0/71 (0%) in the SP+AQ and 0/77 (0%) in the AQ+AS arms respectively. By day 28 the equivalent parasitological failure rates were 4/26 (15%, 95%CI 4–35) in the SP, 18/77 (23%, 95%CI 14–34) in the CD, 1/73 (1% 95%CI 0.001–7) in the SP+AQ and 7/75 (9% 95%CI 4–18) in the AQ+AS arms respectively. After correction by molecular markers for reinfection, the parasitological failure rates at day 28 were 18% for CD, 1% for SP+AQ and 4.5% for AQ+AS; numbers in the SP arm were considered too small to be reliable. Full data are shown in Table 2. Adjusted and unadjusted odds ratios comparing each arm with the best and worst arms (AQ+AS and SP respectively) are shown in Table 3. Relative risks are also shown for comparison with trials which do not use OR. Clinical and parasitological outcomes for AQ+SP and AQ+AS were significantly better than for SP or CD. Although there was a significant parasitological failure rate, both for monotherapy and the combinations the parasitological responses among pregnant women were substantially better than among children under 5 years in the same site 2 years prior to the current study (Fig 2).

**Table 2:** Clinical and parasitological outcomes by days 14 and 28 after treatment.

	SP	CD	SP+AQ	AQ+AS
*Clinically relevant outcomes*				
Number assessed by day 14	24	78	71	77
Clinical failure by day 14 (%).	0	1 (1.3)	0	0
Parasitological failure by day 14 (%).	1 (4)	0	0	0
Adequate clinical and parasitological response (ACPR) by day 14 (%)	23 (96)	77 (99)	71 (100)	77 (100)
Mean haemoglobin (g/dL) at day 14 (SD).	9.1 (1.2)	9.3 (1.1)	8.9 (1.2)	9.1 (1.2)
Median change Hb (g/dL) from baseline (IQR)	−0.2 (−0.8 0.3)	−0.25 (−.85 0.3)	0.10 (−.05 0.4)	−0.3 (−0.6–0.3)
Largest drop in Hb by day 14 (g/dl)	−1.6	−3.2	−3.0	−3.3
Number assessed by day 28	26	77	73	75
Clinical failure by day 28 (%)	1 (4)	11 (15)	0	1 (1)
Parasitological failure by day 28 (%)	3 (12)	7 (9)	1 (1)	6 (8)
Adequate clinical and parasitological response, day 28. (% and 95%CI)	22 (85%, CI 65–96)	59 (77%, CI 66–86)	72 (99% CI 92–100)	68 (91% CI 82–96)
Failures due to recrudescence if those replicating up (to day 28)	0/3	7/9	1/1	2/4
Clinical or parasitological failure rate by day 28 after correction of reinfection.	0 (Numbers small)	18%	1%	4.5%
Failures showing DHFR triple+DHPS double mutation; N = 25 (to day 28)	50.0%	64.3%	0%	66.7%
	(2/4)	(9/14)	(0/1)	(4/6)
Recudescences showing DHFR triple+DHPS double mutation; N = 10 (to day 28)	0%	85.7%	0%	50%
	(0/0)	(6/7)	(0/1)	(1/2)
*Gametocytes*				
Prevalence day 14% (N assessable)	19.1 (21)	25.0 (64)	7.9 (63)	4.7 (64)
Mean AUC of gametocyte density/uL over time, (IQR)	24.4 (4.0–135)	36.2 (11.9–115.5)	20.5 (6.0–63.4)	8.1 (4.0–16.0)

**Table 3:** Difference in parasitological outcomes between the arms- unadjusted and adjusted for age, gestation, initial parasitaemia and haemogobin.

	Unadjusted odds ratios (95%CI)	Sig p	Adjusted odds ratios (95%CI)	Sig p	Unadjusted relative risk ((95%CI))
AQ+SP v AQ+AS	0.13 (0.02–1.1)	0.06	0.13 (0.015–1.1)	0.06	0.15 (0.02–1.2)
AQ+SP v SP^1^	0.08 (0.005–0.7)	0.025	0.06 (0.006–0.6)	0.02	0.09 (0.01–0.76)
AQ+SP v CD^2^	0.046 (0.006–0.36)	0.003	0.046 (0.005–0.36)	0.004	0.06 (0.008–0.43)
AQ+AS v SP	0.56 (0.15–2.1)	0.4	0.61 (0.16–2.4)	0.47	0.61 (0.19–1.9)
AQ+AS v CD^3^	0.34 (0.13–0.86)	0.02	0.36 (0.14–0.94)	0.04	0.40 (0.18–0.90)
SP v CD	0.60 (0.18–2.0)	0.4	0.57 (0.16–2.0)	0.4	0.66 (0.2–1.8)

**Best arm first in each pairwise comparison, without PCR adjustment.**

1 AQ+SP significantly better than SP.

2 AQ+SP significantly better than CD.

3 AQ+AS significantly better than CD.

**Figure 3:**
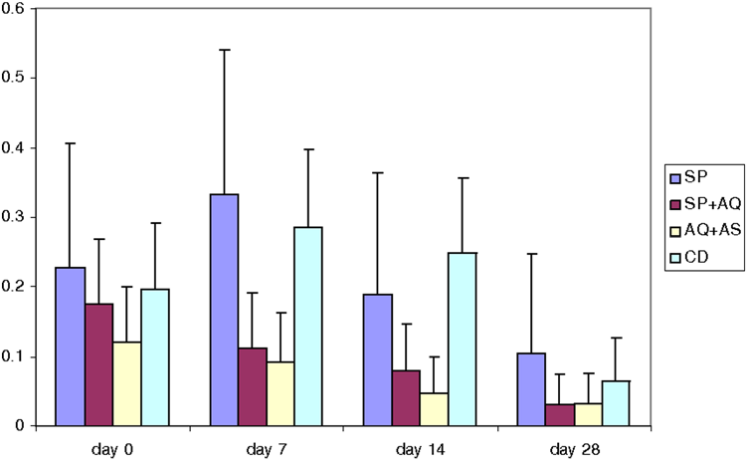
Prevalence of gametocytes, by study drug and day after treatment. SP- sulfadoxine-pyrimethamine. SP+AQ- sulfadoxine-pyrimethamine+amodiaquine. AQ+AS amodiaquine+artesunate. CD- chlorproguanil-dapsone.

At day 14, restricting data to patients with no gametocytes at baseline, 5/16 women (31%) in the SP arm, 12/50 (24%) in the CD arm, 5/54 (9%) in the SP+AQ arm and 3/56 (5%) in the AQ+AS arm were gametocytaemic (Fig 3). The odds ratio of gametocytes in the AQ+AS arm compared to other arms was therefore 0.12 (95%CI 0.02–0.78 p0.004) for SP, 0.18 (95%CI 0.03–7.4 p0.006) for CD and 0.55 (OR 0.08–3, p0.4) for AQ+SP.

There were two maternal deaths during the trial. One woman in the CD arm had mild malaria both clinically and parasitologically when she entered the trial, but developed hyperparasitaemia (>20% parasitaemia) and severe malaria over 48 hours. Review of initial blood films showed that the initial parasite count was correct, but all parasites were synchronous pre-schizonts. She came from a mountain area with little malaria transmission. The second woman in the SP+AQ arm made an initial response but then deteriorated despite clearing her parasites. Consent for determining her HIV serostatus was not given, but other clinical factors suggest it is likely she died from an immunosupression related illness. In neither case was the direct effect of study drugs thought likely to have been the cause, although CD may have failed to stop progression of severe disease in the first case. No other maternal SAEs were recorded; non-severe maternal adverse events are recorded in Table 4. There were minor biochemical, haematological and ECG abnormalities following administration of drugs outlined in Table 5, but no clear patterns except possibly a small increase in prolonged QTC interval of less than 500 milliseconds in the SP+AQ arm; all resolved.

**Table 4:** Adverse events by day 28, Serious Adverse Events (SAEs) and birth outcomes at 6 weeks post-delivery by study drug.

Drug (n)	SP (28)	CD (81)	SP+AQ (80)	AQ+AS (83)
*Non-serious adverse events*				
Nausea/vomiting (%)	3	20	33	35
Abdominal pain	1	6	0	4
Diarrhoea	0	9	0	2
Dermatological, including itching (%)	0	2	10	2
Dizziness	1	1	7	7
Respiratory complaints	0	2	0	3
*Birth outcomes*				
Mean weight at delivery, Kg (SD)	3.0 (0.6)	3.1 (0.5)	3.0 (0.7)	3.2 (0.6)
Median weight of placenta, Kg	0.56	0.56	0.56	0.53
Caesarean sections (%)	1	1	1	3
Minor abnormal birth outcomes (%)	6/26 (23%)	13/74 (18%)	14/75 (19%)	15/79 (19%)
Minor abnormality at 6 weeks	3	7	8	3
Major abnormality at 6 weeks	0	0	0	0

**Table 5:** Biochemical, haematological and ECG changes by drug class.

	SP	CD	SP+AQ	AQ+AS
Median AST day 0 U/l (IQR)	9.7 (8.6–13.4)	9.6 (7.2–12.8)	10.1 (7.8–13.5)	9.2 (7.2–12)
Median AST day 3 U/l (IQR)	9.2 (7.7–12.7)	12.7 (8.1–18)	9.7 (6.8–12.3)	8.8 (6.8–11)
Cases with AST >50 U/l day 3 (concentrations)	0	2 (64.6, 83)	1 (58.3)	0
Platelets×10^9^ L^−1^ day 0 (IQR)	123 (104–171)	165 (112–191)	154 (123–195)	165 (112–191)
Platelets×10^9^ L^−1^ day 3 (IQR)	179 (142–216)	170 (112–198)	171 (144–212)	170 (112–198)
Cases with platelets <50×10^9^ L^−1^ d 3 (absolute counts).	1 (47)	0	0	0
Neutrophils×10^9^ L^−1^ day 0 (IQR)	3.2 (2.3–3.8)	3.1 (2.3–4.1)	3.1 (2.3–4.3)	3.5 (2.3–4.2)
Neutrophils×10^9^ L^−1^ day 3 (IQR)	2.8 (1.3–4)	3 (1.9–3.9)	2.9 (2.2–3.9)	3.1 (2.3–4.1)
Neutrophils <0.5×10^9^ L^−1^ day 3 (count)	0	2 (0.4, 0.4)	1 (0.2)	2 (0.1, 0.4)
ECG QCT interval ms day 0 (IQR)	426 (414–441)	422 (410–441)	428 (411–443)	424 (415–433)
ECG QTC interval ms day 3 (IQR)	422 (411–438)	423 (407–437)	427 (415–445)	426 (411–434)
ECG QTC interval >440 ms	4	18	8	9
ECG QTC interval >500 ms	0	0	0	0

IQR- Inter Quartile Range.

No foetal deaths occurred within 28 days of administration of drugs except in the two women who died. There was one macerated stillbirth in the AQ+AS arm. Other adverse birth outcomes, largely relating to complications related to asphyxia are shown in Table 4, along with caesarean section rates and outcome at 6 weeks following birth. There were 15 stillbirths or deaths within 48 hours of delivery. In the SP arm there was one premature delivery at 27 weeks; the baby died at 32 hours. For CD there was a stillbirth to a 40 year old HIV positive woman, 2 neonatal deaths, one in a twin at 30 weeks, the other in a case of abruption placenta. Two babies died within 24 hours following prolonged labour, and there was one death following obstructed labour. In the SP+AQ arm there was one intrauterine death, two deaths in twins who dies after home delivery, and a child failed to control the neck at 6 weeks secondary to prolonged second stage of labour. In the AQ+AS arm there was a breech birth of a macerated baby at 40 weeks, a stillbirth at term, a intrauterine death and a stillbirth in a twin, the other surviving. Other SAEs at or following birth were: one baby an extra digit each hand; SP+AQ one child with encephalopathy (probably ischemic), one with peupural sepsis, one born with slow reflexes at birth, resolved by 6 weeks, one jaundiced at birth resolved at 6 weeks; AQ+AS one baby hyperpigmented at birth, resolved. Four of the deaths were in twins which had not been identified at admission. There were additionally 3 sets of twins, one in each of the CD, SP+AQ and AQ+AS arms which had normal deliveries.

## Discussion

Balancing the risk-benefit of antimalarial drugs in pregnancy is not easy [33]. Older drugs have a better known safety profile in pregnancy, but parasite resistance to them is likely to be higher than for new drugs and combinations. In this trial, conducted in an area of known moderate to high rates of drug-resistant malaria both to SP and AQ, the combinations AQ+SP and AQ+AS were more efficacious than either SP or CD monotherapy in pregnant women, although probably because the SP arm was small the difference between SP and AQ+AS was non-significant. Dizziness that was common in AQ-based combinations may be due to transient hypotensive tendencies [34]. There is no evidence that either of the drug combinations was less well tolerated than monotherapy, except in minor gastrointestinal side effects. The artemisinin combination AQ+AS was not associated with any detectable increase in adverse birth outcomes when used in the last two trimisters of pregnancy. Malaria in pregnancy is a very serious disease for both mother and fetus; effective treatment is essential. In this area of East Africa the two drug combinations can therefore be recommended for treating proven malaria in pregnancy.

CD had a similar failure rate to SP, a difference from the results of a similar study conducted in children under five years of age in the same area 5 years ago, where CD treated malaria that had failed to respond to SP^22^. It is possible that the dose of chlorproguanil that was used in CD was not high enough to attain adequate therapeutic levels in pregnancy. It was one of two generally used dosing regimens, the other being one based on 2 mg/kg chlorproguanil per day. The lower dose was chosen because of fear of possible risk of drugs in pregnancy, and a higher dose might have proved more effective [35]. Earlier pharmacokinetic studies during pregnancy in Thailand showed that doubling the recommended dose of proguanil was required to attain similar blood drug concentrations in a non-pregnancy state [36], [37]. An alternative is that antifolate resistance has increased still further in this area. With the recent withdrawal of CD following concerns about anaemia with the CDA combination, it is unlikely CD will be available for use in pregnancy; on this evidence it would not be an appropriate drug for use in pregnant women in coastal East Africa at the current dose.

It is encouraging, as seen in Fig 2, that despite the exceptionally high parasite resistance rates to AQ and SP in children in this area of Tanzania, combinations using these drugs are comparatively efficacious in pregnant women, and this is backed up by data from the very different epidemiological setting of West Africa [38]. The current trial is the second time the SP+AQ combination has been shown to be efficacious in pregnant women in Africa, the other being a previously reported trial from Ghana [26]. For policy this should be interpreted with caution in this area; in East Africa this initially efficacious combination rapidly lost its efficacy in children. This, however, makes clear the limitations of using data on drug efficacy in children to derive drug treatment policies in pregnant women, and it strengthens the fact that combinations containing older drugs which are losing efficacy in children may still remain efficacious in pregnancy. This is especially important in the case of IPTp, for which drugs must have a proven safety record as they will frequently be given to women who are not parasitaemic [39], [40]. This means that the risk-benefit is less heavily weighted in favour of using a highly efficacious but potentially teratogenic drug than is the case in parasitologically proven cases.

As in children, the artemisinin-based combinations had a far greater impact on gametocyte carriage than non-artemisinin combinations, even when (as was the case with SP+AQ) the drug combination was itself highly efficacious. The relative immunity which is the likely cause of the different impact on efficacy between pregnant adults and children does not seem so marked for gametocytes. This is potentially important when considering the likely impact of ACTs on transmission, since a substantial proportion of the transmission of malaria is from adults.

Tanzania has a moderate prevalence of HIV in pregnancy, but unfortunately the numbers in this trial are not big enough to answer the question as to which drugs are likely to be most appropriate in HIV-infected pregnant women. There is a complex interaction between HIV and malaria in pregnancy; HIV both increases the risks of side-effects of some drugs and reduces the efficacy of antimalarials [41], [42]. Multi-centre and multi-site clinical drug trials in pregnancy, coupled with HIV testing, are urgently needed to determine drug response in HIV-infected pregnant women with consequent development of appropriate drug policies.

This study adds to the existing data, mostly from studies in Southeast Asia, demonstrating no evidence of teratogenicity when artemisinins are used in the last two trimesters. This is reassuring now that ACTs are being rolled out. It cannot settle the question about the safety of artemisinins early in the first trimester, which is the period that has raised most concern on safety in animal studies. There is no evidence from human studies that artemisinins are teratogenic, but data is still too sparse to rule this out. It may be that non-artemisinin combinations, especially for IPTp, remain a sensible option in some settings for the interim. The investigators decided at the outset of the trial not to study artemether-lumefantrine (Coartem), despite being an excellent antimalarial, because there was no data on safety in African pregnant women. One study conducted in Thailand indicated that the pharmacokinetics of Coartem is deranged in pregnancy [43].

The major limitations of the study are the fact that the size was smaller than anticipated, and that women without typical symptoms of malaria are likely to be under-represented (as they do not present), and women with placental malaria but no peripheral parasites by definition cannot be included. Bias is unlikely to be a major issue as this is a randomised trial, although in smaller trials important random variations between arms can occur.

Despite the fact that the sample size is small for definitive conclusions on safety, it is reassuring both that newer drugs and combinations, including the artemisinin combinations, are tolerable and efficacious in pregnant women in East Africa. In this area, monotherapy with SP or CD for malaria in the last two trimesters of pregnancy, whilst it has antimalarial parasitological failure rates substantially lower than in children, is unacceptably high and should be abandoned. The risks of malaria in pregnancy are too great to continue to use drugs with appreciable parasitological failure rates.

## Supporting Information

CONSORT S1 CONSORT Checklist
(0.06 MB DOC)

Protocol S1 Trial Protocol
(0.16 MB PDF)
