# Supplementary material for: Randomized Trial of Artesunate+Amodiaquine, Sulfadoxine-Pyrimethamine+Amodiaquine, Chlorproguanal-Dapsone and SP for Malaria in Pregnancy in Tanzania
Source: PLoS One. 2009 Apr 8;4(4):e5138. doi: 10.1371/journal.pone.0005138 (PMC2662423; doi:10.1371/journal.pone.0005138)
Supplement: Protocol S1 — Trial Protocol (0.16 MB PDF) [file pone.0005138.s002.pdf]

Trial Protocol (Nov 2003, with modification Oct 2004 highlighted).

Treating malaria during pregnancy: a randomized trial of potential options for treatment in an area of high drug resistance in Tanzania.

This protocol has been approved by ethical committees in Tanzania and the UK.

PRINCIPAL INVESTIGATOR(S):

Dr. TK Mutabingwa, NIMR seconded to LSHTM
Dr. Christopher Whitty, LSHTM

COLLABORATORS AND AFFILIATIONS:

Dr. Kandi Muze (Seconded from Muhimbile)
Dr. Martha Lemnge NIMR
Prof. Brian Greenwood (LSHTM)

PROPOSED STARTING DATE: Nov 2003 DURATION: 2 years

1) SUMMARY

Pregnant women are vulnerable to malaria, with significant implications both for their health and for the pregnancy. Sulfadoxine-pyrimethamine (SP) is currently the first line drug for the treatment of malaria in pregnancy in Tanzania and surrounding countries, but resistance is emerging rapidly. Alternative drugs must be found, and new drugs and drug combinations are being recommended by many for deployment as first line treatment at the point that SP resistance forces a policy change. However, there are few data on the safety and efficacy of these combinations in pregnant women. This randomised trial aims to assess efficacy and safety, including birth outcome, in pregnant women with malaria in the second or third trimesters. A total of 900 pregnant women will be randomised either to standard treatment (SP) or to one of three potential drugs, or drug combinations recently recommended by a WHO expert panel. These will be SP-amodiaquine, chlorproguanil-dapsone (Lapdap), and amodiaquine-artesunate. Primary outcome will be treatment failure at day 28. Secondary outcomes will include maternal side effects, foetal viability and birth outcome.

2). Background.

Malaria in pregnancy is an important preventable cause of maternal and perinatal morbidity and mortality (Brabin 1991). In areas where infection with *Plasmodium falciparum* is endemic, immunity to malaria develops over the first few years of life, and older children and adults rarely suffer severe complications of infection. An exception is during pregnancy, when risks of severe maternal anaemia (Shulman 1996) and low-birthweight (Steketee 1996a) are increased by malaria infection. The risks are greatest in the first pregnancy and decrease with increasing gravidity (McGregor 1984). Malaria infection in pregnancy in these situations is often asymptomatic (Steketee 1996b). For this reason, preventative strategies such as chemoprophylaxis or intermittent treatment with antimalarials have been recommended in pregnancy (WHO). It is also now recommended that case management of severe anaemia includes antimalarials.

However, all pregnant women are also at increased risk of clinical disease compared with non-pregnant women (Diagne 1997). Clinical disease during pregnancy needs prompt effective treatment, as even seemingly uncomplicated disease can result in foetal loss and can progress to severe disease. Particular dangers of severe malaria in pregnancy (which are more common and more severe in non-immune women and possibly women with HIV infection), include high fever, which can precipitate a premature delivery, hypoglycaemia, severe anaemia, pulmonary oedema and maternal death (Meek 1988, Nosten 1991). Severe anaemia is a particular problem in pregnant women in Africa who were, before pregnancy, semi-immune.

In Tanzania, current first line treatment for all patients with uncomplicated malaria is sulfadoxine-pyrimethamine (SP). This has the advantage of being a one-dose treatment that can be given as an outpatient. The only current alternative treatment for pregnant women is quinine which needs to be given for 7 days, and usually therefore requires in-patient treatment. Quinine is also often associated with unpleasant side effects. For many women without severe disease, outpatient management with an effective antimalarial should suffice. However, there is an urgent need to find an effective alternative to SP due to increasing levels of resistance.

Drug resistance: Increasing chloroquine resistance has necessitated changing the first line antimalarial drug to sulfadoxine/pyrimethamine (SP) in many countries in Africa. Emerging resistance to SP is increasing at an alarming rate. Drug sensitivity tests conducted recently by the East Africa Network for Monitoring Antimalarial Treatment (EANMAT) have shown SP-treatment failure rates of ≥ 25% at several sites (EANMAT, 2001a), the threshold level of drug resistance recommended by the WHO for changing an antimalarial drug. Nonetheless, options of alternative drugs are limited because the few available drugs are either not recommended for use as monotherapy (artemisinins), are expensive (mefloquine, Malarone), have inappropriate dosage schedule for outpatient use (quinine), or have an uncertain safety profile in pregnancy.

Drug resistance within the study area: The study will be done in Muheza district, where there is already considerably expertise in running clinical trials successfully under the direction of Dr. Mutabingwa, currently seconded to the Gates malaria programme from NIMR. Muheza district participated in the study on "Malaria chemosuppression during pregnancy" by Mutabingwa *et al.* (1993) and the LSHTM has long-standing research links in this area. The lowlands of Muheza district experience hyperendemic to holoendemic malaria (Clyde 1967; MARAMAP 2000). More than 95% of the malaria infections are caused by *Plasmodium falciparum.* Resistance of *P. falciparum* to SP is relatively high in the district, providing a strong rationale for using this site to test new combinations that might be adopted nationally. In a recent study in Muheza, the 14-day parasite clearance by SP was 55% (Mutabingwa *et al* 2001). In this study, re-treating malaria clinical episodes emanating from parasitaemia that failed first treatment with SP resulted in higher SP-resistance (61%). In contrast, a 3-day course of chlorproguanil-dapsone (LapDap) cleared all but 7% of the parasitaemia. EANMAT data from Mkuzi Health Centre

(Muheza district), showed that the combined early and late treatment failure rate to amodiaquine was 11% (7/64) compared to 34% (23/68) for SP (EANMAT, 2001b). Malaria parasites in this area are still highly sensitive to quinine, which is used as the third line drug or for treating severe and complicated malaria. Quinine therapy either alone for 7 days or as a 3-day course plus a single curative dose of SP cleared parasitaemia by day 7 in all patients (n=200) admitted to Muheza Hospital in 1997/98 with severe and complicated malaria (Mutabingwa *et al-in preparation*).

Existing treatment policy: The National malaria treatment policy until August 2001 was to use chloroquine to treat and prevent malaria in pregnant women attending Maternal and Child Health (MCH) clinics. The new policy, operational since 1st September 2001, is to use SP for first line treatment of malaria and for Intermittent Preventive Therapy (IPT) in pregnant women. There is high SP-resistance in the district (Mutabingwa *et al* 2001)although published data is from studies in children, so cannot be automatically extrapolated to pregnant women. HIV seroprevalence among blood donors is 11% in Muheza district (Acting Medical Superintendent of the hospital-*personal communication 2002*), although blood-donor seroprevalence tends to overestimate population seroprevalence in East Africa. HIV is a co-infection shown to impair the efficacy of IPT with SP during pregnancy. For these reasons the current use of SP alone during pregnancy is questionable.

The health leadership for Muheza district and Tanga region recognizes the threat posed by malaria, in particular antimalarial drug resistance and the urgent need to search for and institute practicable control measures for malaria during pregnancy. The Medical Superintendent for Muheza Designated District Hospital (DDH) and the Regional Medical Officer for Tanga region agree with the aims of this study and believe that it will help to develop an evidence based treatment policy.

The trial and its justification. Recently, WHO proposed four different drug regimes for treating uncomplicated malaria. A number of trials in children suffering from falciparum malaria have established elsewhere that all proposed drug combinations are efficacious and safe in East Africa. Extrapolating from trials in children to pregnant women is not, however, reliable either for efficacy (because of differences in pharmacokinetics and immunity) or for side-effects and effect on birth outcome. The relative efficacy and safety of these combinations of drugs during pregnancy are poorly documented. Clinical trials of combinations of antimalarial drugs in pregnant women are urgently needed in order to develop evidence based treatment regimes for malaria during pregnancy.

3. OBJECTIVES OF THE STUDY

The objectives of this study are to assess the therapeutic efficacy and safety of SP as the current first line drug, and three other potential alternative combinations in treating uncomplicated falciparum malaria during pregnancy, in an area with appreciable levels of SP resistance and rising HIV seroprevalence. Specifically the study will compare the clinical and parasitological response to and the side effects of the following drug regimes:

(a) SP;
(b) SP + amodiaquine [SPAQ];
(c) Chlorproguanil+dapsone (Lapdap);
(d) Amodiaquine + artesunate [AQAS]

4. TRIAL DESIGN

4.1 Primary end point

The primary end-point of the trial will be treatment failure by day 28. This will be defined as:-

Any of:-
1) a need for rescue treatment due to clinical deterioration defined by altered sensorium, convulsions, persistent vomiting, renal impairment, respiratory distress, a fall in Hb below 7g/dl, or in cases where initial haemoglobin was less than 9 g/dl a drop of 20% from the starting Hb, at any time during admission;
2) persistence of fever with parasitaemia on day 3;
3) increasing parasite load on day 3;
4) failure to clear parasites on day 7;
5) rescue medication for recurrent malaria before day 28;
6) slide parasite positivity at day 28.

4.2 Secondary endpoints

Secondary endpoints will include the following:-

1. Incidence of foetal death during treatment, defined as absence of foetal heartbeat assessed by Doppler;
2. Hypoglycaemia requiring treatment;
3. Parasite recrudescence or re-infection on day 14;
4. Parasite clearance on day 3;
5. Level of recovery of haemoglobin on day 14;
6. Fever clearance time;
7. Incidence of perinatal and neonatal mortality, assessed 4-6 weeks after due date of delivery;
8. Clinically apparent neonatal abnormality, assessed 4-6 weeks after due date of delivery;
9. Placental malaria
10. Preterm delivery
11. Other adverse events during treatment.

4.3 The trial

1. This will be a randomized controlled trial with four arms;
2. The slide reader assessing the primary endpoint will be blind to treatment allocation, and analysis will be performed by intention-to-treat;
3. Direct Observed Therapy (DOT) of all doses of study regimens will be employed;
4. Drug doses packed in blister packs, labelled with the patient number will be used where possible.

5. SELECTION AND WITHDRAWAL OF PATIENTS

Pregnant women with mild-moderate, slide proven, falciparum malaria will be recruited from the Antenatal wing (ANC) of the Maternal and Child Health (MCH) clinic at Muheza (Teule) Hospital. Pregnant women from Muheza Township and surrounding villages attend this clinic. Nurses attending the clinic will interview all febrile women and women with a recent (past 24 hours) history of fever and those with a probable diagnosis of malaria will be referred to the study team. Those with signs/symptoms of mild-moderate anaemia will also be referred. All referrals will be re-interviewed and examined by the Medical Officer of the study team to exclude concomitant infection(s). Duplicate thick and thin blood smears will be made, Giemsa stained at pH 7.2, and examined microscopically. The consent form will be administered to those meeting the inclusion criteria and they will be enrolled upon consenting.

5.2 Inclusion criteria

A pregnant woman who has:-

either a positive blood smear for *P.falciparum* with at least 1000 asexual parasites/µL in an asymptomatic woman
or any of the following symptoms within 2 days prior to consultation: history of fever; headache, vomiting, chills/rigors, and/or any of the following signs: temperature $\geq 37.5^0$C and $<39.5^0$C, Hb≥5 and <9 g/dl) together with *P.falciparum* parasitaemia at any density

and (in both cases) the following:

1. has no exclusion criterion (see below);
2. is 14-34 weeks pregnant on the day of attending the ANC clinic or OPD;
3. has a viable foetus, defined by presence of foetal heartbeat by sonicaid or pinnard (foetal heartbeat is not heard until 14 weeks);
4. is able to take study drugs by the oral route;
5. is able to attend stipulated days for follow up clinic and provide specimens;
6. gives informed written or witnessed verbal consent to participate by herself, and also through her parent/guardian if aged <15 years (in conformity to Tanzania Law).

Those > 34 weeks are excluded because they are close to term and may deliver during the 28 day f/u period.

5.3 Exclusion criteria

Exclusion criteria include:-

1. severe and complicated forms of malaria (as defined by WHO);
2. pregnancy in the first trimester;
3. a mixed plasmodial infection;
4. complicated pregnancy e.g. signs/symptoms of toxaemia of pregnancy;
5. 2 or more abortions or stillbirths;
6. presence of concomitant disease masking assessment of the response to treatment ;
7. an intake of drugs contraindicated in pregnancy e.g. tetracycline, cotrimoxazole or a macrolide antibiotic;
8. an intake of drugs with effective antimalarial activity within the last 2 weeks[*]
9. significantly abnormal baseline haematology (except anaemia) or clinical chemistry parameters e.g. laboratory evidence of renal impairment (serum creatinine >2 mg/dl) or of hepatitis (alanine aminotransferase ALT>5 times upper limit of normal);
10. previous participation in the study: Women having a second episode of malaria after completing the 28-day follow up will have details recorded and offered quinine but not be re-enrolled.
11. multiple gestation pregnancies eg twins
12. mother aged 38 years or above

Patients with malaria who do not enter the trial because they fulfil an exclusion criterion will be treated in the optimum way decided by the attending physician. In general, those with severe disease will receive parenteral quinine whilst those with mild disease will receive SP.

5.4 Withdrawal criteria

Withdrawal criteria will include:-

[*] prior chloroquine ingestion will not be an exclusion criterion. Drug ingestion in the previous 2 weeks will be acertained by patient interview as full testing for all locally available drugs with antimalarial activity is not feasible.

1. withdrawal of consent or non-compliance with assigned study regimen;
2. appearance of other species of Plasmodium;
3. vomiting within one hour after re-dosing;
4. protocol violation.

If it is necessary to withdraw a patient during the treatment phase, administration of the study drug will be discontinued. If the patient is still parasitaemic, quinine will be given as a rescue therapy unless there are clinical reasons to use another drug. For withdrawals outside the treatment phase the team will carry out all the safety and efficacy assessment measurements that would have been carried out at the next scheduled visit and the same will apply at delivery (unless the patient is lost to follow up). In such cases the study conclusion page in the Clinical Report Form will be completed and the study medication records updated. Patients withdrawn from the study will continue attending routine antenatal care provided by the MCH clinic. Finger prick blood smear for microscopy and Hb. will be done at every withdrawal. If rescue treatment is given, the blood smear will be checked again on day 7 post-treatment to ascertain parasitological clearance. Patients withdrawn before day 7 and not classified as treatment failures will be replaced.

5) Patients who fail on treatment will be treated with rescue treatment and counted as treatment failures (see above).

6. TREATMENT OF PATIENTS

6.1 Study regimens

Choice of drugs: On 5th April 2001, WHO/RBM convened a meeting of experts to discuss and decide on possible alternative chemotherapeutic drugs to SP for the treatment of malaria in non-pregnant patients in areas of SP resistance. Using a pre-set selection criteria, the meeting arrived at 4 possible alternatives and based on currently available information, ordered the combinations in order of current preference as:-
artemether-lumefantrine (Co-artem)> amodiaquine + 3-days artesunate ($AOAS_3$)> SP + 3-days artesunate ($SPAS_3$)>SP + amodiaquine (SPAO) (WHO 2001).

This trial will investigate two of these four options. The investigators consider that a randomized trial of the sort proposed is probably not the appropriate vehicle for investigating artemether-lumefantrine at this stage. Although animal toxicity studies showed no teratogenic effects at doses $\geq$ 100 mg/kg of the combination (although some embryotoxic effects were seen), and lumefantrine alone shows no evidence of mutagenicity at doses up to 1000 mg/kg (Novartis 2000), there are no existing studies involving lumefantrine-containing regimens in pregnant women. Of the other regime not tested as it now looks unlikely that the SP/artesunate combination will be deployed in areas where SP resistance is significant.

SP/amodiaquine and amodiaquine/artesunate have been chosen as useful alternatives to S/P which would be affordable locally. Officials from the Ministries of Health (MoH) in the sub-region who participated in the 13th EANMAT Secretariat meeting (June 2001) in Dar-es-Salaam, ranked these combinations as highly appropriate for the sub-region and worth testing (*Minutes, EANMAT Sec13 in Dar-es Salaam*). S/P has been assessed in pregnancy (Verhoeff *et al* 1998, Parise *et al* 1998, Shulman *et al* 1999) and is now recommended for intermittent treatment in pregnancy.

Both artesunate and artemether have been given to many pregnant women (often inadvertently), and current published data demonstrate no evidence of human teratogenicity (McGready *et al* 1998). Follow up of over 539 women seen in Thailand who received an artemisin for treatment

of malaria in pregnancy showed no adverse effects on the mother or on her baby (McGready *et al* 2001). Following WHO recommendations (1995) artemisinin derivatives have been used for malaria treatment during the second and third trimesters of pregnancy where multi-drug resistance is likely. Whilst most of the data from treatment doses is from Asia, 287 pregnant women in the Gambia given a single dose of SP-artesunate had no increased rate of adverse birth outcomes (Deen et al 2001). Recent meetings of toxicologists and clinicians convened by the WHO (July 2002) reviewed all available data. Whilst there is some evidence of fetal toxicity in some animals early in pregnancy, there was felt to be no evidence from human studies contradicting proceeding with studies with artemisinin containing drugs in the second 2 trimesters of pregnancy (report in progress).

Amodiaquine also has been given to many hundreds of women in pregnancy in Africa and elsewhere, either inadvertently or deliberately, and studies have demonstrated no teratogenicity (Thet Naing *et al* 1988), although formal safety data are sparse. The WHO recommends it can be used for treatment of malaria in pregnancy (1995). Although formal side-effect studies on these combinations (SPAQ AQAS) have not yet been conducted, hence this proposal, there is sufficient data from pregnant women to justify the combinations chosen in a randomized trial which actively looks for side-effects and birth outcome in women where the drug is given after the first trimester.

Chlorproguanil-dapsone has recently proved effective as monotherapy in children in the Muheza district (Mutabingwa *et al* 2001). It is likely to be a relatively cheap antimalarial, and may be useful in pregnancy used as monotherapy, in combination therapy or in intermittent therapy in pregnancy. Whilst experience with chlorproguanil-dapsone in pregnancy is limited to use with a single dose, where it appeared safe (Keuter *et al* 1990), proguanil has been recommended for use as the safest antimalarial in pregnancy for many years. Experience with dapsone treatment during pregnancy used in Hansen's disease (Kahn 1985, Lyde *et al* 1997) and in other pregnancy related conditions (Lush *et al* 2000) is reassuring. There are theoretical reasons to be cautious of antifolates in pregnancy, but in the case of S/P (and co-trimoxazole) there has so far been little data to suggest they pose any hazard in the second and third trimesters of pregnancy (WHO 1986).

Study drugs will be purchased or sourced from reputable sources with Good Manufacturing Practice (GMP). Dosages will be based on the body weight (kg) of the patient and the schedule will be as below.

(a) SP (sulfadoxine 25mg/kg stat)[SP];
(b) SP (sulfadoxine 25mg/kg stat) + Amodiaquine (10mg/kg x 3 days) [SPAQ];
(c) Chlorproguanil-dapsone (1.2 mg/kg and 2.4 mg/kg respectively 3 days) [Lapdap]
(d) Amodiaquine (10mg/kg x 3 days) + Artesunate (4mg/kg x 3 days) [AQAS]

6.2 Regime allocation

Women will be randomized in blocks of ten (each block with 1 SP group and 3 each of the other three groups). Random numbers between 1 and 800 will be generated by the computer (EPI INFO) programme and allocated to regimens until 80 numbers are in the SP+ placebo group and 240 each in the three other treatment groups. On consenting to participate, patients will be registered numerically in the enrolment log as they present. The registration number will determine the drug regimen.

6.3 Clinical treatment and investigations.

All women enrolled will be admitted to the research ward (to be built by the GMP) for the first three days for DOT and to monitor clinical response and adverse events. Drugs will be administered by nurses employed by the project. After each administration, the patient will be observed for 45-60 minutes to ascertain drug retention. The dose will be repeated if vomiting occurs within the observation period, and any further vomiting will cause withdrawal from the study and treatment with parenteral quinine. Patients will be treated for symptoms by standard treatment e.g. paracetamol for fever. Women will also continue to receive routine antenatal medicaments of iron supplements, folic acid, and Tetanus Toxoid (TT) given by the MCH. Drugs with an antimalarial activity will not be permitted; if these are essential, the subject will be withdrawn.

Vital signs (temperature, pulse) will be monitored eight hourly during the treatment period and once daily during the remaining period of hospitalisation. Blood pressure will be measured once daily during hospitalisation. Patients will be interviewed using a "clinical assessment form" check list which includes: weakness, chills/rigors, headache, myalgia, dizziness, abdominal pain, anorexia, nausea, vomiting, diarrhoea, palpitations, insomnia, pruritus, coughing, and tinnitus. Blood glucose will be measured on admission and regularly through treatment by glucose stix testing.

Foetal viability (presence of foetal heartbeat) will be monitored daily during admission using a Doppler machine, and at each follow-up visit.

6.4 Adverse events (other than treatment failure)

a) If a patient is unable to tolerate the trial medication the reason for discontinuation will be recorded in the Clinical Record Form as an "adverse event" and alternative rescue medication initiated.

b) Any clinical or laboratory abnormality which in the opinion of the senior attending clinician might be or is likely to be due to the study medication will be recorded as an adverse event.

Quinine at a dose of 10-mg/kg body weight will be the rescue medication for those who require it.

Adverse events will be classified according to:

Severity	a) non-severe, b) severe
Causality in relation to medication	a) not related, b) unlikely to be related, c) possibly related, d) probably related, e) most probably related, f) insufficient data/information to assess

Subjects with an AE will be followed up until the condition has disappeared or stabilized.

6.5 Laboratory investigations

Parasite count will be performed once every morning during admission, repeated on days 7, 14 and 28, and on any other day(s) of complaints. Counts will be made against 200 white blood cells (WBC) on a thick blood smear and quantified per μL using the baseline WBC count of 8,000 and multiplying each by 40.

Blood for haematology and clinical chemistry, together with urine for routine urinalyses will be obtained on admission, on the last day of study drugs (day 3) and at day 7. Further testing will not be performed unless clinically indicated.

Haematological tests will include: haemoglobin, and where appropriate total and differential white blood cell count and platelet count
Biochemical parameters will include creatinine, total bilirubin, alanine aminotransferase (ALT) and albumin.

HIV testing.

There is a possibility that HIV co-infection could significantly alter the efficacy of antimalarial drugs in pregnant women- certainly this seems to be the case for SP [Kisumu]. It may also affect birth outcome. It is hoped that in a randomised design the number with HIV will be balanced between the groups, which based on the blood donor seroprevalence is unlikely to be a large number in any group. Knowing the HIV serostatus of women enrolled would however be ideal, as at a minimum it would allow for stratification by HIV status (negative, positive or not known).

The Ministry of Health in Tanzania has recently recommended that all pregnant women should be offered HIV testing after pre-test counselling. In Teule hospital nevirapine is currently available for HIV positive women around the time of delivery. Major efforts are being made in Teule hospital to provide structured support for those found HIV positive, and home-based palliative care for those with advanced HIV disease. The study team will follow national and hospital guidelines for women enrolled in the study, and offer testing after pre-test counselling to all women, as for any other pregnant women as part of routine antenatal care. Where they agree to testing the results will be recorded if they consent to this. Agreeing to HIV testing will not, however, be a pre-condition of entry to the trial. The study team considers that anonymous testing would be unethical in the context of this study. Currently take-up of HIV testing is low in Muheza district despite a very positive attitude to it from Teule senior staff and those who accept HIV testing may well be systematically different in terms of HIV risk from those who refuse. This study is therefore unlikely to be the ideal platform to examine the interactions between HIV and antimalarial drugs in pregnancy.

6.6 Follow up to day 28.

On discharge from the ward, patients will be requested to attend follow up clinics at the hospital on days 7, 14, 21 and 28 and at any time they have a clinical problem before day 28. Village Health Workers will visit them daily at home up between discharge and day 7, and if they do not attend follow up. During clinic visits the progress will be reviewed, complaints recorded, and laboratory specimens (blood smear, 2-3 mls blood for full blood picture, drug assays etc) collected when appropriate. Temperature and pulse will be measured and recorded. At the end of each clinic, members of the study team will follow all “no show up cases” to their homes to record reasons for not showing up, explain the importance of adhering to stipulated follow up days and to collect clinical and laboratory variables. During daily home visits VHWs will elicit any history of drug intake outside the study team and record progress using a simple standardised questionnaire, collect blood smears from febrile women and submit it to the study team for microscopy. The VHW will refer sick women to the study team for review and expert management.

7 Follow up at birth and after delivery.

(a) Birth outcome will be recorded for all study mothers who deliver in Teule hospital (currently between 70 and 80% of those who attend antenatal clinics). This will include maternal mortality, perinatal mortality, maternal complications, and whether the new-born baby has any clinically apparent abnormalities.

(b) Patients will be followed up at home by VHWs on day 7 after birth for kernicterus. Previous studies have demonstrated that following up at home works well in Muheza district.

(c) Patients will be encouraged to bring their babies to Teule for routine immunisation one month after birth, and assessed for birth outcome at that point. At 6 weeks after due date, all mothers (whether giving birth in Teule or not) who have not attended will be followed up at home. Assessment at that time will include birth outcome, or a serious problem since birth such as kernicterus, and the baby will examined for congenital abnormalities. In the case of a neonatal or maternal death a history will be taken from the main carer or any healthcare workers involved to try and ascertain the cause of death. Whenever the mother has moved from the area all possible efforts will be made to ascertain birth outcome.

7 Sample size and duration of study.

NOTE ADDED Oct 2004- the sample size calculation in italics is the original one. There is a revision following slow recruitment due to local control measures. This is in normal font and blue.

Sample size has been calculated based on treatment failure as defined in section 4.1. If the response of pregnant women to antimalarial treatment is similar to that of children in Muheza, the proportion of women with treatment failure by day 28 in the SP+Placebo group will be at least 45%, the parasitological clearance failure rate among sick children at the study site (Mutabingwa et al., 2001). A Cochrane data review of studies of amodiaquine in Africa showed a day 14 clearance failure rate on amodiaquine alone was 17% (Olliaro and Mussano, 2000) leading to a theoretical failure rate for SP+Amodiaquine of at least 8% (0.45 x 0.17) if there is no interaction between these drugs. In the Gambia, day 14 failure rate with SP+Artesunate was 1.6% (von Seidlein et al 2000). Assuming the 28-day failure rate of 4% with artesunate alone, the failure on amodiaquine+artesunate is estimated at at least 1% (0.17 x 0.04). In this study, it is assumed that the best treatment would achieve 5% failure rate. We will consider anything up to 10% failure rate to be equivalent for public health purposes.

Allowing for a 10% defaulter rate approximately 40 women per group would be sufficient to detect a five fold decrease in the risk of treatment failure in the three combination groups compared to the SP group. However this sample size is insufficient for comparing the risk of treatment failure between the different combinations. It is important to compare the efficacy of SP+ amodiaquine versus the combination including artesunate as the latter combination is likely to be more expensive and hence advantage over the former needs to be defined. Thus, in order to detect a 4 fold difference in treatment failure between SP+Amodiaquine and amodiaquine+artesuante group (eg 8% vs 2%) with 95% precision and 80% power, we will enrol 80 women in the SP+placebo group and 240 women in each of the other three groups.

Based on the sample size of 80 women on SP alone and 240 in each of 3 combination regimens while allowing for 10% loss to follow up, a total of 900 women will be enrolled in the study. According to records at MCH for year 2000, a total of 6,880 pregnant women (approximately 20 per day) attended the antenatal clinic, of which an average of 6 per day had complaints. We assume that 3 of the sick 6 will have falciparum malaria and that 2 of them will consent to participate. Therefore, it will take 450 working days to enrol 900 women and the duration of the study is estimated to be 2 years.

The sample size should be adequate and sufficiently robust to detect common side effects in pregnancy. Rare side effects can only be detected in large Phase 4 and post-marketing surveillance studies. This study should provide information which can allow such studies to be undertaken.

Oct 2004. Vigorous measures to protect pregnant women in the district from malaria on a general background of reduced transmission of malaria in this area have fortunately led to substantial reductions in the number attending the antenatal clinic with clinical malaria. The investigators reviewed the data from other sites, and it is clear there is still likely to be a critical shortage of data on antimalarials in pregnancy from Africa, so a trial even with an ability to detect this relatively large difference is likely to be of considerable utility to policymakers. A revised sample size has been calculated to detect a difference at day 28 from 1% (the best likely failure rate in any arm) and 15% (above which no drug could be deployed). This gives a sample size of 72 in each arm when α was .05 and β 0.8. This would be capable of detecting common, but not rare, adverse events. The trial will continue on this basis due to the public health importance of this question.

9 Stopping rules/discontinuation criteria (finalised after meeting of the DSMB)

Parasite clearance: The study has to balance the Tanzania government's recent policy to use SP as first line drug treatment against a high SP-resistance in Muheza district. We assume that around 45% of women in the SP group will need escape medication. Since all women will be admitted to the study ward, the escape medication will be administered on time and complications arising from the use of a relatively ineffective drug should be minimal. However, if the proportion of women needing escape medication is ≥ 40% in any arm of the study, we will discontinue that arm when 50 women are enrolled which will give a confidence limit of 25 -55% for the estimated treatment failure rate.

Side effects. All serious possible side-effects and foetal deaths will be faxed to the DSMB statistician. All other adverse events will be forwarded. The DSMB (see section on ethics) will decide on whether one arm, or the whole study, needs to be terminated. Suggested guidelines for this following the first meeting of the DSMB is that the DSMB would take note following one SAE in an arm, and consider whether an arm needed to be terminated after 2, but would review in the light of data art that stage. The DSMB wishes to be informed if there is a fall in haemoglobin of >4 g/dl, OR where haemoglobin falls below 5 g/dl and these will be treated as a potential SAE, but since most such drops will be secondary to the natural history of the disease these will be noted and discussed, but treated differently from other SAEs.

10. PCR for recurrent parasitaemia

Blood for PCR will be collected from all patients at enrolment and on the day of re-appearance of parasitaemia. PCR analysis of parasite genes will be performed only in patients with re-appearance of parasitaemia in order to differentiate between re-infection and recrudescence. Blood spots will be collected on glass fibre membranes and DNA extracted using standard techniques. The polymorphic repetitive regions to be amplified by nested-PCR are block 2 of msp1 and block 3 of msp2 (Snounou *et al,* 1993). In a first reaction, polymorphic sequences of these genes will be amplified using primer pairs to their conserved regions. Using the template of the first PCR reaction, allele-specific primer pairs will be used to test for the presence of the allelic variants from the MAD20, RO33 and K1 families of the msp1 and FC27 and 3D7/IC of the families of the msp2. PCR products will be analysed by gel electrophoresis. Amplification patterns of the various allelic families in DNA samples from day 0 will be compared to other samples from the same patient who was parasitaemic on or after day 14 post-treatment. If the allelic family (ies) amplified on day 0 are identical in size to those amplified from subsequent episode, then the patient is classified as carrying a recrudescent infection. If however the pattern of amplification is different for any allelic family, then it is assumed that this is a new infection.

11. Data analysis.

The primary outcome will be by treatment defined as having at least one dose of study drug. A secondary analysis will be by intention to treat defined as being enrolled into the treatment log and assigned a number. Treatment failure as defined in section 4.1 is the primary outcome.

Other pre-planned analyses are:

- Fetal viability;
- Birth outcome 6 weeks after due date;
- Parasite clearance rate at day 3;
- Parasite recrudescence or reinfection rate by day 14;
- Fever clearance rate (time);
- Level of recovery of Hb on day 14;
- Adverse events;
- Relapse confirmed by PCR.

Planned stratified analyses are by parity and HIV serostatus.

12 Capacity strengthening.

Provisional plans for capacity strengthening locally have been dicussed with the Acting Medical Superintendent, Teule Hospital, and the Director, NIMR-Amani. Provisional plans include the following (this is not an exhaustive list).

Building and equipping of a ward at Teule Hospital. The ward will be potentially multi-use, and can be used for clinical studies or patient use for pregnant mothers or children. This ward has been considered by the Board of Governers of Teule Hospital, and they consider it very desirable for their long-term needs if there is a period it is not necessary for clinical studies.

Training in clinical trials for Hospital staff and the possibility of secondments for NIMR clinical staff.

Teaching ward rounds and clinical involvement in the hospital by senior trial staff.

Training in PCR for NIMR staff, either locally or in London depending on what is appropriate.

Training in clinical chemistry for laboratory technicians.

Improved clinical laboratory infrastructure.

A clinical platform for pharmacological and immunological studies by NIMR and LSHTM staff.

13 REFERENCES

1.Brabin BJ (1991) *The risks and severity of malaria in pregnant women. Applied Field Research in Malaria Reports*. World Health Organization, Geneva.
Clyde DF (1967). *Malaria in Tanzania*. Oxford University Press, Oxford.
2. Diagne N, Rogier C.,Cisse B and Trape JF (1997) Incidence of clinical malaria in pregnant women exposed to intense perennial transmission. *Trans. R. Soc. Trop. Med. Hyg*. **92,** 166-170.
3. Deen JL, von Seidlein L, Pinder M, Walraven GE, Greenwood BM (2001). The safety of the combination artesunate and pyrimethamine-sulfadoxine given during pregnancy.*Trans R Soc Trop Med Hyg*. 2001 **95**(4):424-8.4. EANMAT (2001a). Monitoring antimalarial drug resistance within National Malaria Control Programmes: the EANMAT experience *Trop. Med. Int. Health*, **6**: 1-8, October 2001 (in press)
5. EANMAT (2001b). Treatment of falciparum malaria in East Africa: sulfadoxine-pyrimethamine (SP) monotherapy is not the way forward, EANMAT New Letter (Chairman's Column), Vol. 4, Issue 2, May 2001.
6. Kahn G. (1985)Dapsone is safe during pregnancy. J Am Acad Dermatol. **13**(5 Pt 1):838-9.
7. Deen JL, von Seidlein L, Pinder M, Walraven GE, Greenwood BM. The safety of the combination artesunate and pyrimethamine-sulfadoxine givenduring pregnancy.Trans R Soc Trop Med Hyg. 2001 Jul-Aug;95(4):424-8.
8. Keuter M, van Eijk A, Hoogstrate M, Raasveld M, van de Ree M, Ngwawe WA,Watkins WM, Were JB, Brandling-Bennett AD. (1990) Comparison of chloroquine, pyrimethamine and sulfadoxine, and chlorproguanil and dapsone as treatment for falciparum malaria in pregnant and non-pregnant women, Kakamega District, Kenya. *BMJ*. **301**(6750):466-70.
9. Lush R, Iland H, Peat B, Young G. (2000) Successful use of dapsone in refractory pregnancy-associated idiopathicthrombocytopenic purpura.*Aust N Z J Med*. **30**(1):105-7.
Lyde CB. Pregnancy in patients with Hansen disease.Arch Dermatol. 1997 May;133(5):623-7. 10. McGregor IA (1984). Epidemiology, malaria and pregnancy. *Am. J Trop Med. Hyg.* **33**: 517-525.
11. McGready R, Choo T, Cho JJ et al (1998). Artemisin derivatives in the treatment of falciparum malaria in pregnancy. *Trans. R. Soc. Trop. Med. Hyg.* **92**: 430-3
12. McGready R, Cho T, Keo NK, Thwai KL, Villegas L, Looareesuwan S, White NJ,Nosten F. Artemisinin antimalarials in pregnancy: a prospective treatment study of 539 episodes of multidrug-resistant Plasmodium falciparum.Clin Infect Dis. 2001 Dec 15;33(12):2009-16.
13.Meek, S.R. (1988) Epidemiology of malaria in displaced Khmers on the Thai-Kampuchean border. *Southeast Asian. J. Trop. Med. Public. Health*, **19,** 243-252.
14. Mutabingwa et al (1993). Malaria chemosuppression during pregnancy. I-VI 45.
15. Mutabigwa TK, Nzila A, Mberu E *et al.* (2001). Chlorproguanil-dapsone for treatment of drug-resistant falciparum malaria in Tanzania. *Lancet* **358**: 1218-23..
16. Nosten, F., ter-Kuile, F., Maelankirri, L. *et al* (1991). Malaria during pregnancy in an area of unstable endemicity. *Trans. R. Soc. Trop. Med. Hyg*. **85,** 424-429.
17. Olliaro P and Mussano P (2000). Amodiaquine for treating malaria. Cochrane database system review 2000; 2:CD000016.
18. Parise, M.E., Ayisi, J.G., Nahlen, B.L., *et al.* (1998) Efficacy of sulfadoxine-pyrimethamine for prevention of placental malaria in an area of Kenya with a high prevalence of malaria and human immunodeficiency virus infection. *Am. J. Trop. Med. Hyg*. **59**, 813-822.
19. Shulman, C.E., Graham, W.J., Jilo, H. et al (1996). Malaria is an important cause of anaemia in primigravidae: evidence from a district hospital in coastal Kenya. *Trans. R. Soc. Trop. Med. Hyg*. **90**, 535-539.
20. Shulman, C.E., Dorman, E.K., Cutts, F. *et al.* (1999) Intermittent sulfadoxine-pyrimethamine to prevent severe anaemia secondary to malaria in pregnancy: a randomised placebo-controlled trial. *Lancet*, **353,** 632-636.
21. Snounou G, Viriyakosol S, Zhu XP et al (1993). High sensitivity of detection of human malaria parasites by the use of nested polymerase chain reaction. *Mol.Biochem. Parasitol*. **61**:315-20
22. Steketee, R.W., Wirima, J.J., Hightower, (1996a) The effect of malaria and malaria prevention in pregnancy on offspring birthweight, prematurity, and intrauterine growth retardation in rural Malawi. *Am. J. Trop. Med. Hyg*. **55**: 33-45.
23. Steketee, R.W., Wirima, J.J., Slutsker, L *et al* (1996b), Comparability of treatment groups and risk factors for parasitemia at the first antenatal clinic visit in a study of malaria treatment and prevention in pregnancy in rural Malawi. *Am. J. Trop. Med. Hyg*. **55(1 Suppl),** 17-23.
24. Thet Naing, Hla Win, Yin Yin New (1988). Falciparum malaria and pregnancy: relationship and treatment response. *Southeast Asian J. Trop. Med. Pub. Heal.* **19**: 253-8
25. Verhoeff FH, Brabin BJ, Chimsuku L et al (1998). An evaluation of the effects of intermittent sulfadoxine-pyrimethamine treatment in pregnancy on parasite clearance and risk of low birthweight in rural Malawi. *Ann. Trop. Med. Para*. **92**: 141-50.
26. von Seidlein L, Milliagan P, Pinder M *et al* (2000). Efficacy of artesunate plus pyrimethamine for uncomplicated malaria in Gambian children: a double blind, randomised, controlled trial. *Lancet* **355**:352-7.
27. WHO (1991). *Research in Malaria Reports*. World Health Organization, Geneva.
28. WHO (1995). *Management of Uncomplicated Malaria and the Use of Antimalarial Drugs for the Protection of Travellers. Report of an informal consultation*. World Health Organization, Geneva.
29. WHO (2000). *WHO expert committee on malaria 20th report*. World Health Organization, Geneva.

30. WHO (2001). Antimalarial drug combination therapy. Report of a technical consultation. World Health Organization, Geneva.

ETHICAL ISSUES

Discuss ethical aspects of the proposal.

1) Approval by the local Institutional Review Board (IRB) or Ethical Committee (IEC)

This protocol was submitted to Ethical Committees of the National Institute for Medical Research in Tanzania and has been approved. It has been submitted to the London School of Hygiene and Tropical Medicine Ethics Committee and has been approved.

2) Subject information and Informed Consent

a) Each woman will receive written information (in Kiswahili) about the study, which will also be explained in the presence of the witness when applicable. Sufficient time will be allowed for the woman to decide if she wishes to join the study;
b) Women will be allowed to enquire about details of the study and any question regarding the study will be answered;
c) The PI will ensure that the consent form has been signed and dated by the participant, attending clinician and/or witness where applicable;
d) In the case of a patient who cannot read, an independent literate witness will attend the information session, sign and date the consent form on behalf of the patient;
e) In case of protocol amendments, the PI will ensure that the consent form is also revised to reflect the approved amendments.

3) All drugs used in the study have been used for treating malaria in pregnancy previously (see section 6.1 for further details), although not necessarily in the combinations proposed in this study.

4) An independent Data and Safety Monitoring Board (DSMB) will be set up. This will receive reports of all adverse events, and of treatment failures to ensure it is safe to continue the trial. Exact terms of reference and provisional stopping rules will be determined in consultations between the Committee and the trial investigators.

5) This study will contribute to capacity strengthening;

a) through improved infrastructure at Teule hospital (deemed appropriate with authorisation given by the Governors of the Hospital and by the Ministry of Health);
b) through provision of additional clinical services and training within the hospital for the duration of the study;
c) provision of an infrastructure that can support additional projects.

Appendix 1

Drug administration and collection of monitoring variables of mother

Time and events schedule

	**Treatment with study drugs**			**Follow-up through D 28**				**At delivery**	**At the time of treatment failure**
Study Day	**0 Screening/ Baseline**	**1**	**2**	**7**	**14**	**21**	**28**		
Written Informed Consent	✓								
Inclusion/Exclusion Criteria	✓								
Demography	✓								
Medical History	✓								
Physical Examination	✓	✓	✓	✓	✓	✓	✓	✓	✓
Fetal viability	✓	✓	✓	✓	✓	✓	✓		✓
Vital Signs (BP, PR, RR) Temperature	✓[a]	✓[a]	✓[a]	✓	✓	✓	✓	✓	✓
Assessment of Clinical Symptoms of Malaria	✓	✓	✓	✓	✓	✓	✓	✓	✓
Adverse Event Review	✓	✓	✓	✓	✓	✓	✓	✓	
Concurrent Medication Review	✓	✓	✓	✓	✓	✓	✓	✓	
Malaria Finger-prick blood smears	✓[b]	✓[b]	✓[b]	✓	✓	✓	✓	✓	✓
Blood Sample for Parasite DNA	✓								✓
HIV test	✓								
Clinical Chemistry	✓			✓[c]					
Haematology	✓			✓					
Routine urinalyses	✓			✓					
Treatment with study drug	✓	✓	✓						
Placental and Newborn investigations [d]								✓	
Assessment of new-born[e]									

a. Temperature to be monitored every 12 hours until the temperature falls below 37.5 ^{o}C and remains normal for at least 24 hours.
b. Blood smear done once daily during hospitalization, at follow up visits, and at any time of complaint of fever.
c. If abnormal, follow up assessment will be done once weekly until the values are normal.
d. Placental blood smear, cord blood smear, newborns weight (kg), and Dubowitz assessment if day 28 is around delivery time.
e. Weight and maturity assessment if day 28 is around delivery and women delivers at hospital (study site)

Note: BP = blood pressure, PR = pulse rate, RR = respiratory rate

Monitoring of child at birth and one month is outlined in the text.

Appendix 2 Extracts from SOPs.

Quality control and assurance will be instituted through written standard operating procedures (SOPs) to assure that the trial is conducted and that data are generated, recorded, and reported in compliance with the protocol, good clinical practice (GCP), and the applicable regulatory requirements. Prior to enrolment, the clinical monitor and the PIs will review the protocol and all trial related procedures, information on the study regimens, procedures for obtaining informed consent, procedures for completing CRFs and for reporting AEs. The monitor will make regular visits, at which, information in the CRFs will be verified against the SD for accuracy and completion. Also, the monitor will review the informed consent procedure, drug accountability and storage, trial documents and trial progress. The clinical monitor will verify that the investigator follows the approved protocol and/or amendments (if any), and will discuss any problem/issue for improvement.

All women enrolled will be admitted to the research ward (to be built by the GMP) for the first three days for DOT and to monitor clinical response and adverse events. Patient demographic data and socioeconomic background will be collected. Clinical symptoms/signs and haematological profile will be recorded in a semi-structured enrolment form with a standardized checklist of symptoms and signs. At discharge, an identity card will be issued to facilitate follow up, prevent drug administration outside the study team and to exclude them from the study if they develop a second episode of malaria after completing the 28 day follow up.

Drugs will be administered orally by nurses employed by the project. After each administration, the patient will be observed for 45-60 minutes to ascertain drug retention. The dose will be repeated if vomiting occurs within the observation period, and any further vomiting will cause exit from study and treat with parenteral quinine.

If a patient is unable to tolerate the trial medication and treatment is discontinued, the reason for discontinuation will be recorded in the Clinical Record Form as “adverse event” and rescue medication initiated.

At study conclusion, the Clinical Monitor will return all unused medications to the sponsor, after a careful check and authorization.

Although the proposed study regimens are considered safe, there is need to look for any unlikely serious adverse event (SAE).

A SAE shall be reported within 24 hours by telephone, fax, or e-mail to the safety monitoring board. Thereafter, the PI will submit a written report describing the SAE, its onset, date of onset, type, duration, severity, cause-effect relationship to the administered drug(s), outcome and measures taken.

SAEs will be recorded in the CRFs, evaluated and discussed at the conclusion or termination of the study.

Personal data of patients will be kept confidential.

The agreement covering the use of data or analysis will be documented in writing together with the written informed consent for participation in the trial.

The PI will retain study material for an agreed period. Destruction of study material thereafter, will require a joint written authorization from interested parties.

All trial data will be recorded on the CRFs in English. Only the PI and a few authorized co-workers (as on the list of Authorized Signatory Form) will make entries.

Entries will be legible in black or blue ballpoint pen. Corrections should be made by drawing a single line through the original entry, entering the new value with initials and date next to the new entry. Completed CRFs will be dated and signed by the PI.

All clinical data will be entered into medical records. The clinical and laboratory results will be transcribed into the CRFs. SDs will be kept in a separate file from CRFs.

The clinical monitor will review all completed CRFs for completeness and accuracy before data entry into computer. Entry will have checks to identify errors and protocol violations. Request for clarifications and/or corrections on errors/violations will be sent to the PI. Analysis will be carried out after all queries have been addressed and database locked.

The PI will ensure that all study drugs are received, stored and documented. All study medications will be stored at room temperature under lock and key at the study site.

The PI will issue the study medications to the “dispensing nurse” of the study team. The dispensing nurse will account for all used and unused drug supplies. A drug accountability list will be kept in the investigator’s file. Remaining medications will be kept in the original blister packs. The nurse will keep blister packs with or without remaining drugs for inspection and validation against the drug accountability list

The PI will maintain a) a drug receipt form to confirm that supplies have been received b) a list of all drugs given to and returned by the dispensing nurse and c) a record of all unused drugs. Study drugs will only be used according to the study protocol; any discrepancy should be explained.

All personal demographic identification and clinical data/history will be recorded in the medical records to constitute the Source document (SD**).** The clinical data and laboratory results will be transcribed in the Clinical Report Form (CRF). The SDs and CRFs will be kept separately at the office of the PI.

The day of first treatment is day 0. Day 14-cure rate is the primary end point of therapeutic efficacy. It is the proportion of treated patients with no asexual parasitaemia on day 14, and who did not receive rescue medication.

An efficient medication should increase the haemoglobin level by at least 1 g/dl within 14-28 days of starting treatment, assuming no other causes for blood loss. To avoid group differences in taking iron/folate supplements, the study team will ensure that all participants receive standard routine care.
